# Metropolitan Hospital Sunday Fund, 1888

**Published:** 1888-08-04

**Authors:** 


### THE METROPOLITAN HOSPITAL SUNDAY FUND, 1888.

THE meeting of the Council of the Hospital Sunday Fund on Tuesday should be memorable in the history of the metropolitan hospitals. This is so because a larger sum has been collected on the present occasion than ever before, a fact which is mainly, if not entirely, due to the support given to the movement by the leading morning and evening metropolitan papers. Apart from the special efforts made by this journal and the *Lancet*, the *Times* devoted many columns of its space day by day throughout "the hospitals week" to the advocacy of hospitals. Other papers gave valuable, though spasmodic, support. Amongst these must be gratefully mentioned the *Morning Post*, the *Daily Telegraph*, the *Daily News*, the *Echo*, the *Pall Mall Gazette*, and the *Star*. The managers of the London hospitals have always laboured under a debt of obligation to the metropolitan editors; but this indebtedness has been heightened and deepened by the proceedings this year in connection with Hospital Sunday. Few ordinary people recognize the difficulties under which the London editor labours in the busy season of the year at the height of which the Hospital Sunday collections are made in the metropolis. Every day columns and columns of matter have to be thrown aside for want of room; and we believe it will be some satisfaction to those members of the Press who have so nobly helped the hospitals this year to learn that their efforts have reduced the cost of collecting the money by some £500, as compared with previous years; but even without this diminished cost of collection the amount available for distribution would have been larger than it has ever been before. Such a result will, we hope, encourage the metropolitan press to recognize and support the Fund day by day throughout "the hospitals week" for the future, now the *Times* has set so good an example. In any case, we thank all those editors who have contributed to the very encouraging success which has undoubtedly been obtained by the Hospital Sunday Collections during 1888.

THE report of the Distribution Committee of the Metropolitan Hospital Sunday Fund is remarkably clear and courageous. Public opinion has probably been in advance of the critical action of this Committee for some few years past, and its report this year will therefore be welcomed. To-day it is our pleasant duty to congratulate Sir Sydney Waterlow, the founder of Hospital Sunday in London, who from the first has laboured assiduously with a noble devotion to secure the success of the Fund, and so to make permanent the voluntary principle of hospital support. His labours and those of his colleagues on this committee have not been inconsiderable, and the thanks of all concerned are due, and will be given in no grudging spirit to them. A word of commendation and encouragement must be given to Mr. Henry N. Custance, the secretary of the Fund, and to Mr. Owthwaite, his assistant. Both these gentlemen have acquired an experience in hospital accounts which is invaluable to the Council, and the way in which the statistics are prepared and presented is worthy of all praise.

WE are sorry that through a misunderstanding we last week published the report of the Distribution Committee and a list of the awards made by the Committee of the Metropolitan Hospital Sunday Fund *collections*. This report should not have appeared until after the meeting of Council on Tuesday. Though no harm has been done, we regret the inadvertence which led to the misunderstanding in question. The attention of editors of the metropolitan papers is directed to the fact that the *Charity Record* copied the information from last week's HOSPITAL and sent it round in slips—a course its editor has taken on more than one recent occasion. Will they kindly make a note of the fact for future guidance?

